# Accelerated Postero-Lateral Spinal Fusion by Collagen Scaffolds Modified with Engineered Collagen-Binding Human Bone Morphogenetic Protein-2 in Rats

**DOI:** 10.1371/journal.pone.0098480

**Published:** 2014-05-28

**Authors:** Xinglong Han, Wen Zhang, Jun Gu, Huan Zhao, Li Ni, Jiajun Han, Yun Zhou, Yannan Gu, Xuesong Zhu, Jie Sun, Xianglin Hou, Huilin Yang, Jianwu Dai, Qin Shi

**Affiliations:** 1 Orthopedic Department, the First Affiliated Hospital of Soochow University, Suzhou, P.R. China; 2 Orthopedic Institute of Soochow University, Suzhou, P.R. China; 3 Orthopedic Department, the Second Affiliated Hospital of Soochow University, Suzhou, P.R. China; 4 Division of Nanobiomedicine, Suzhou Institute of Nano-Tech and Nano-Bionics, Chinese Academy of Sciences, Suzhou, P.R. China; 5 State Key Laboratory of Molecular Developmental Biology, Institute of Genetics and Developmental Biology, Chinese Academy of Sciences, Beijing, P.R. China; Texas A&M University Baylor College of Dentistry, United States of America

## Abstract

Bone morphogenetic protein-2 (BMP-2) is a potent osteoinductive cytokine that plays a critical role in bone regeneration and repair. However, its distribution and side effects are major barriers to its success as therapeutic treatment. The improvement of therapy using collagen delivery matrices has been reported. To investigate a delivery system on postero-lateral spinal fusion, both engineered human BMP-2 with a collagen binding domain (CBD-BMP-2) and collagen scaffolds were developed and their combination was implanted into Sprague-Dawley (SD) rats to study Lumbar 4–5 (L4–L5) posterolateral spine fusion. We divided SD rats into three groups, the sham group (G1, n = 20), the collagen scaffold-treated group (G2, n = 20) and the BMP-2-loaded collagen scaffolds group (G3, n = 20). 16 weeks after surgery, the spines of the rats were evaluated by X-radiographs, high-resolution micro-computed tomography (micro-CT), manual palpation and hematoxylin and eosin (H&E) staining. The results showed that spine L4–L5 fusions occurred in G2(40%) and G3(100%) group, while results from the sham group were inconsistent. Moreover, G3 had better results than G2, including higher fusion efficiency (X score, G2 = 2.4±0.163, G3 = 3.0±0, p<0.05), higher bone mineral density (BMD, G2: 0.3337±0.0025g/cm3, G3: 0.4353±0.0234g/cm3. p<0.05) and more bone trabecular formation. The results demonstrated that with site-specific collagen binding domain, a dose of BMP-2 as low as 0.02mg CBD-BMP-2/cm^3^ collagen scaffold could enhance the posterolateral intertransverse process fusion in rats. It suggested that combination delivery could be an alternative in spine fusion with dramatically decreased side effects caused by high dose of BMP-2.

## Introduction

Low back pain caused by instability of spine is a common disease as the population ages and life styles change. Various factors, such as trauma, tumor, deformity, and degenerative disease, may play prominent roles in spine instability [1], [2]. Spinal fusion is one of the most popular procedures to correct unstable parts of the spine. In the US alone, there are approximately 500,000 spinal fusion surgeries performed annually to recover biomechanical stability, and the numbers are increasing [3], [4]. Spine fusion has become one of the most common surgical interventions for spine reconstruction.

Since their introduction by Albee and Hibbs in 1911 for the treatment of disseminated tuberculosis of the spine, the materials for spine fusion have evolved tremendously [1]. Autogenous iliac crest bone graft has long been considered the “gold standard” used in spinal fusion procedures because its characteristics, such as osteogenesis, osteoinduction, and osteoconduction, are ideal for promoting fusion from a biological perspective [3]. But a series of complications accompanied by autogeneous bone grafting are inevitable. In addition, the limited source of autogenous iliac crest bone graft largely restricts its application to patients, especially the elderly and children [5]. Allogeneic bone is another possible source of the materials. Although allograft bone may be obtained in greater quantities, the problems of complex processing, low biocompatibility and the risk of disease transfer also hinder its applications [6]. Consequently, tissue engineering products have been considerated as the favorable alternative strategy.

Much of attention has been focused on composite materials for spine fusion because of their advantages of biocompatibility, safety, operability. Collagen is the most abundant protein in the body and is also one of the most important components of extracellular matrix. Scaffold manufactured with collagen has shown its excellent biocompatibility and biodegradability [7]. Furthermore, as a carrier, collagen scaffold can provide physical support for cells and also appropriate incorporation sites for mineral substances and cytokines [7]. A number of applications with collagen scaffolds have been extensively accepted in clinic [4], [6], [7].

Bone morphogenetic protein-2 (BMP-2) is a potent osteoinductive cytokine that plays a critical role in bone regeneration and repair. Both basic researches and clinic trials have demonstrated its repair efficacy in bone fractures, nonunion and defects [7]. Its safety in clinical application has also been well studied. In 2002, BMP-2 was approved by American Food and Drug Administration (FDA) to apply to spine fusion for patients [8]. However, some recent clinical reports mention that 30% of BMP-2 was released in burst upon initial implantation because conventional collagen materials have no specific binding affinity to BMP-2. Quick degradation and unsustainability have also been reported as a consequence of burst release [6]. The others found cancer risk showing an upward trend after the doctors used a high dose of BMP-2. These findings raise concerns that this growth factor may act as a cancer promoter [9]. To avoid of the side effects of high dose of BMP-2 in vivo, a collagen binding domain (CBD) has been added to the N-terminal of BMP-2 (CBD-BMP-2), which consequently can enhance the binding of BMP-2 to collagen scaffolds and results in a remarkably sustained release action [10], [11], [12].

To achieve better therapeutic effect and to avoid the local or systemic adverse effects in vivo, here we developed collagen scaffolds from bovine aponeurosis and implanted the collagen scaffolds incorporated with CBD-BMP-2 in a rat spine fusion model and explored the feasibility of this delivery matrices for clinical application.

## Materials and Methods

### Preparation of Collagen scaffolds

Collagen scaffolds were prepared followed a well-established method as previously described [13], [14]. In brief, fresh bovine aponeurosis was separated and its muscles, connective tissues and fats were removed. Next, the cellular components and soluble proteins from aponeurosis were extracted, freeze-dried and made into granular shape. The materials were sterilized by 15 kGy Co^60^ irradiation and weighted. The morphology of the scaffolds was taken photographs by the scanning electron microscope (FEG-SEM, XL-30, Philips, Holland).

### Preparation of CBD-BMP-2

The fusion protein CBD-rhBMP-2 consists of a 6×his purification tag, a collagen binding domain (WREPSFCALS) and mature human BMP2 fragment. It was prepared following the steps in the references [10], [11]. A reconstructed BMP-2 DNA sequence cloned by PCR procedure was linked to a bacterial expression plasmid pET-28a (Novagen, Germany), and then was transfected into the BL21 (DE3) strain of E.Coli. The bacteria were cultured and proliferated in the container at 200 rpm and then induced with 1mM isopropyl β-D-thiogalactopyranoside (IPTG) to express recombinant protein at 37°C for 4 h. The target protein was separated as inclusion body from the lysate of the E.Coli, collected by centrifugation, and purified using chromatography. After being renatured, the fusion protein was concentrated by ultrafiltration. After the activity of CBD-BMP-2 was assessed, the protein was freeze-dried and weighted. 2hrs before the implantation, CBD-BMP-2 was dissolved in normal saline and then loaded into the Collagen scaffolds at 4°C (0.02mg CBD-BMP-2/cm^3^ scaffold).

#### The biological activity of the CBD-BMP-2 in vitro

The MC-3T3-E1 (the Cell Bank of the Chinese Academy of Sciences, Shanghai, China) were used to evaluate the osteoinduction activity of CBD-BMP-2 and commercial BMP2 (East China Pharmaceutical Group Co. LTD, Hangzhou, China). Cells were seed at a density of 2×10^4^ cells/well in 24-well plates and maintained in α-MEM containing 10% FBS for 24h. Then, cells were treated with the osteoblast inducing medium supplemented with 0.5%FBS and CBD-BMP-2 or commercial BMP-2 for additional 3 days. After washed with PBS, the treated cells were lysed with 0.1% TritonX-100/PBS and repeatedly frozen/thawed for three times to disrupt the cell membranes. Alkaline phosphatase (ALP) activities were determined using an alkaline phosphatase reagent kit (ANASPEC, Fremont, CA, USA) and Protein concentrations was measured by the Bicinchoninic Acid (BCA) protein assay reagent (Santa Cruz Biotechnology, USA) [9], [11].

#### The binding and release assay of CBD-BMP-2 in vitro

The collagen scaffolds were sterilized by 15 kGy Co^60^ irradiation, and divided into 10 with an equal volume (8mm^3^). CBD-BMP-2 and commercial BMP-2 (East China Pharmaceutical Group, Hangzhou, China) solutions with an amount of 5 µg (10 µl/scaffold) were added to sterility eppendorf tube and incubated at 37°C for 2 h. Then all the tubes were extensively washed with PBS, and the unconjugated proteins were measured by the Bicinchoninic Acid protein assay reagent [9], [15].

The pre-treated scaffolds were divided 2 groups: the commercial BMP-2 group (n = 5), and the CBD-BMP-2 group (n = 5), were placed respectively in tubes containing 100 µl PBS at 37°C. At various time points (6h, 12h, 24h, 4d, 7d), the scaffolds was removed and the released protein amount was determined by using the Bicinchoninic Acid protein assay reagent (Santa Cruz Biotechnology, USA) [16], [17].

### Construction of rat Lumbar 4-Lumbar 5 (L4-L5) posterolateral spine fusion model

All experimental procedures were performed following the Guide for the Care and Use of Laboratory Animals of the US National Institutes of Health (NIH), and were approved by the Animal Research Committee of Soochow University (No. 20120406–022).

60 male Sprague-Dawley rats (150±27g, Shanghai Slac laboratory animal Co. Ltd, Shanghai, China.) were divided into 3 groups randomly: the sham group (G1, n = 20), the collagen scaffold group (G2, n = 20) and collagen scaffold with CBD-BMP-2 group (G3, n = 20). The posterior-lateral lumbar spine fusion technique was performed as previously described [3], [6], [7], [18]. The animals were anesthetized with intraperitoneal injection of 3% pentobarbital (50mg/kg of body weight), and placed on the operating table in the prone position. A vertical incision was made in the skin and muscle tissue was dissected. Two separate paramedian incisions were made 3 mm from the midline in the lumbar fascia, and then the transverse processes of L4 and L5 were exposed. After decorticated with a high-speed burr, the transverse processes of G2 and G3 were implanted with collagen scaffolds (5mm×3mm×10mm/side) on both left and right sides or with CBD-BMP-2 (3 µg/side) separately, while the control group was untreated. The fascial and skin incisions were closed with a 4–0 absorbable suture after implantation. The surgery was completed by the same person. All animals survived without nerve injury during the surgical procedure. All rats were housed in separate cages and fed rat chow, water and antibiotics to avoid infection ad libitum, and were monitored for general health on a daily basis. There were also no abnormal behavior appearing after the surgical procedure and no death due to infection or other reasons. After 16 weeks, all rats were sacrificed and samples were collected for further research.

### Manual palpation

Manual palpation is an easy and specific way for assessing rat postero-lateral spinal fusion [1], [19], [20], [21]. 16 weeks after surgery, all rats were killed and the spines were collected and the segments from T12 (Thoracic) to sacrum were preserved. After removing soft tissues around the bone masses and facet joints, the explanted lumbar spines for intersegmental motion were blindly tested by three independent orthopedists manually. Only the bilateral fusion without any left or right motion was considered a successful fusion. Any motion detected between either the transverse processes or the facets, or unilateral fusion was regarded as a fusion failure. The spines of all rats were recorded as motion, partial motion or no motion. Motion and partial motion were considered as not fused and no motion as fused.

### X-Ray analysis

At 8 and 16 weeks after surgery, posteroanterior radiographs were taken for each spine (full digital molybdenum targeted photographic scanner, Giotto image MD, Italy). Three independent orthopedists assessed the fusion between the L4 and L5 transverse processes in each rat. If the transverse processes were fused completely, there were a quantity of bone bridges appear and no gap could be found [22], [23]. Unilateral or incomplete fusion was considered as a partial fusion and the lack of bone bridges indicated a failed case, which is scored via the following system: 0 =  no osteogenesis(bone filling less than 25% of the area); 1 =  slight osteogenesis without fusion(bone filling 25% to 50% of the area); 2 =  osteogenesis with partial fusion(bone filling 50% to 90% of the area); 3 =  osteogenesis with complete fusion (bone filling more than 90% of the area)[7], [13].

### Micro-CT analysis

The L4 and L5 of the rats were scanned using micro-CT (Skyscan 1176, Belgium) in the spine position at 8 and 16 weeks, and high resolution scanograms (9–20 µm) were obtained (resolution:18 µm, Source Voltage:65KV, Source Current:385µA, Rotation Step:0.7°). The selected images of L4 and L5 were used for 3D reconstruction, meanwhile we chose a cylinder with 1mm diameter, 1.8mm thick (100 continuous slices) in the image to analyze the bone mineral density (BMD) in the fusion area. Well-known standard substances offered by the factory (Skyscan, Belgium) were used to quantify the BMD.[7], [18], [24].

### Hematoxylin-eosin (H&E) staining

After the explanted spines were collected, they were fixed in 10% formaldehyde solution with a volume that was nine times that of the spine tissues. Bone tissues were decalcified in 5% EDTA solution for 4 weeks, and then embedded in paraffin. The specimens near the transverse processes were sliced sagittally at the level of the transverse process. The sections were stained with hematoxylin and eosin (H&E). Images were recorded by fluorescence microscopy (Axio imager. M1, zeiss, Germany). Histologically, the spine fusion could be observed in the presence of the bony trabeculae from one transverse process to another [7], [25].

### Statistical analysis

The software Statiscal Package for the Social Sciences (SPSS) 13.0 (Chicago, IL, USA) was used to analyze the experimental data. Quantitative data were presented as means ± standard deviation. For the results of radiographic and manual palpation, three groups were compared by rank-sum test or nonparametric *chi*-square test. For the continuous data of BMD and release assay, we used ANOVA in one group at different time points and *t* test or parametric *chi*-square test for different groups at same time point, and any *p*-value less than 0.05 was considered to be statistically significant.

## Results

### Collagen scaffolds were prepared in porosity

**Figure 1:**
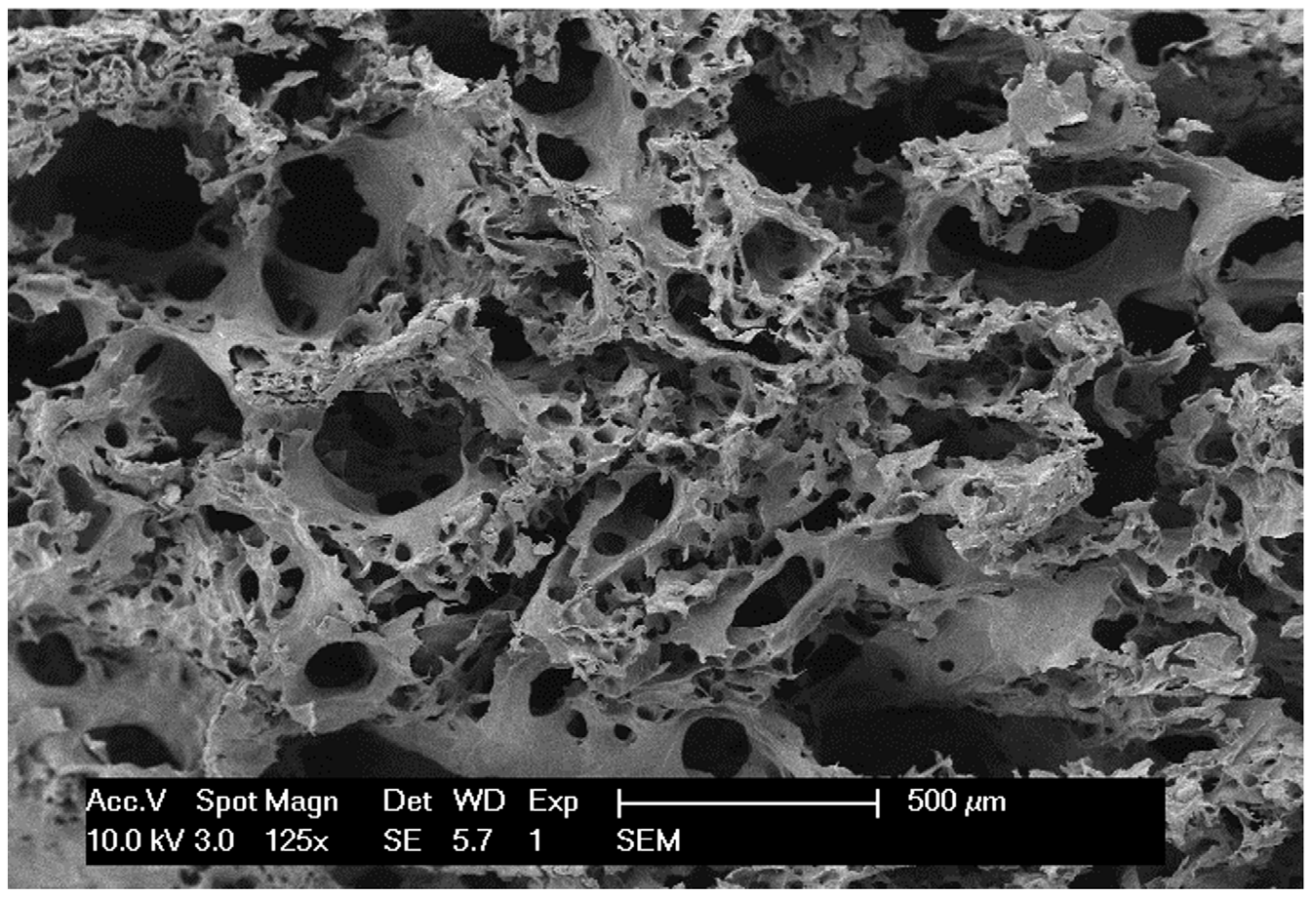
The morphology of the processed collagen scaffolds. This granular scaffolds were taken photographs under the scanning electron microscope (125×). The micropores size range from 100 µm to 300 µm.

As we expected, the collagen scaffolds were prepared from fresh bovine aponeurosis and characterized by a proper porosity (65%). The pore size ranged from 100 µm to 300 µm (Fig.1)[11], [12].

#### The biological activity of the CBD-BMP-2 in vitro

**Figure 2:**
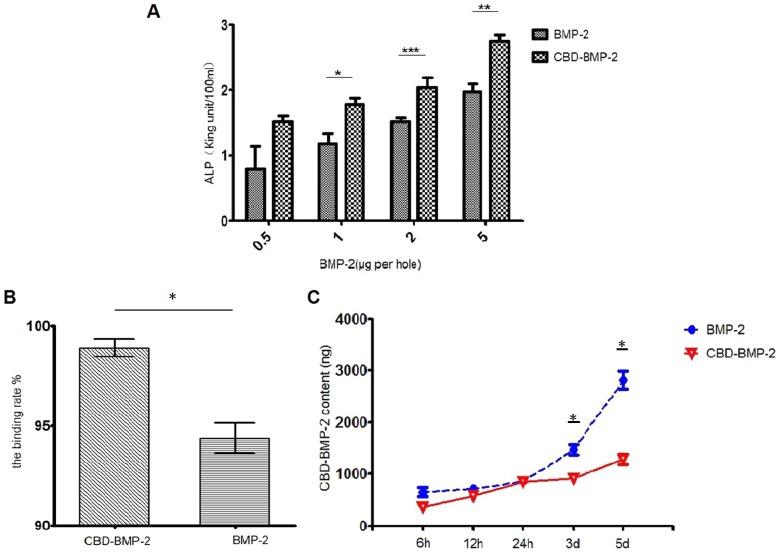
The release profile and bioactivity assay of BMP-2 in vitro. A: The CBD-BMP-2 shows a higher biological activity than the commercial BMP-2 (*:*p*<0.05; **:*p*<0.01; ***:*p*<0.001;). B: The binding rate of CBD-BMP-2 to the collagen scaffolds is higher than that of commercial BMP-2(*p*<0.05). C: The releasing amount and rate in the commercial BMP-2 group was higher than those of the CBD-BMP-2 group.(*, Compare to the control group, *p*<0.05).

The biological activities of the CBD-BMP-2 and commercial BMP-2 were tested by the ALP activity assay using the MC-3T3-E1 cells. After incubating for 3 days with CBD-BMP-2 or commercial BMP-2 over a range of 0.5–5 µg, we found that ALP activities were increased significantly in a dose-dependent manner. And there is a statistical differences in 1, 2, 5 µg/well between the two groups, while no difference in 0.5 µg/well. It shows that the CBD-BMP-2 has a slight higher inductive osteogenesis potential than the commercial BMP-2 (Fig.2A).

#### The binding and release assay of CBD-BMP-2 in vitro

Collagen scaffolds in the CBD-BMP-2 solution resulted in a binding rate of 98.89±0.26% 2hrs after immersion. It showed statistically significant difference with the collagen scaffolds in the commercial BMP-2 solution, whose binding rate was 94.37±0.53% (Fig.2B). The release profiles of CDB-BMP-2 and the commercial BMP-2 are displayed in Fig.2C. At the initial stage (less than 6h), both of them showed slightly burst release. But the cumulative drug release in CBD-BMP-2 was significantly lower than that of commercial BMP-2. There still is a lower released protein content in CBD-BMP-2 group within 24 hours. After 24 hours, the releasing amount and rate in the commercial BMP-2 group was higher than the CBD-BMP-2 group. It indicated that this sustained system could significantly slow down the release of the BMP-2 in vitro.

### Spine fusion occurred in G2 and G3 group by manual palpation

Manual palpation for fusion evaluation was performed by three orthopedists blindly. The results are shown in Table 1. G1 group showed no or partial spine fusion and still reserved the ability of scoliosis. It indicated that no fusion happened without any external factors in the process of rats' recovery. In G2 group, only a few fused spines could be found. But all spines in G3 group were assessed as fused by manual palpation. There were significant differences among three groups. The biological advantages of collagen promoted spine fusion and the incorporation of CBD-BMP-2 at a level of 3 µg/side increased this promotion.

**Table 1:** Assessment of postero-lateral fusion via manual palpation (n = 10 per each group).

	manual palpation (Number)		
	G1	G2	G3
Motion	10	0	0
Partial motion	0	4	0
No motion	0	6	10

There were significant differences among three groups (G2 *vs* G1,*p*<0.05; G3 *vs* G1, *p*<0.05; G3 *vs* G2, *p*<0.05). It indicated that both collagen scaffolds and the CBD-BMP-2-loaded collagen scaffolds can promote a postero-lateral fusion in rats and the latter presented a higher fusion efficiency.

### New bone formation was observed by X-Ray and Micro-CT

**Figure 3:**
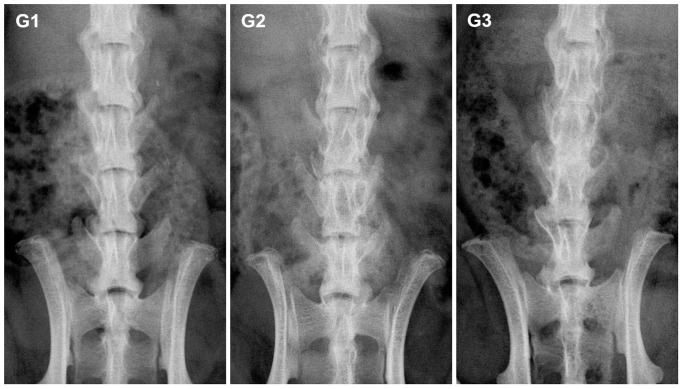
X-Radiographs of SD rat spines in vivo. Representative images of rats in each group are shown (coronal view). 8 weeks after the implantation, the spines appeared fused in G3, partially fused in G2 and no fused in G1.

**Figure 4:**
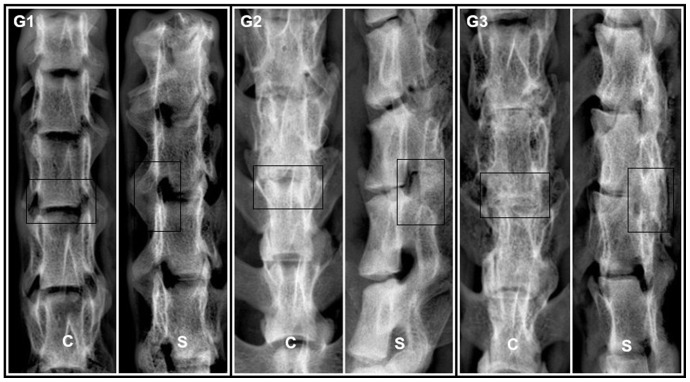
X-radiographs of SD rat spines in vitro. The Representative images showed that visible fusion occurred in both G2 and G3 groups at 16 weeks after the implantation (coronal view (C) and sagittal view (S)). The intervertebral space in G3 was hid under the new bone. A great quantity of new bone could be found and the processus transversus were joined to form a whole from a posterior lateral aspect between L4 and L5. There was a similar, but slightly imperfect result in G2.

X-ray was used to study the fusion between the L4 and L5 transverse processes, as shown in Fig. 3. According to the orthopedists' judgment, no bony bridge was detected in G1 (Fig. 3, G1). In contrast, a significant amount of bone bridges were found both in G2 and G3 groups, and the bone bridges in G3 group were much denser (Fig.3, G2, G3). After the rats were sacrificed, radiographs of all the SD rat spines were obtained in vitro (Fig. 4) and the same results were observed as from the images obtained in vivo. Differences were significant among three groups (Table 2, score: G1 = 0.8±0.133, G2 = 2.4±0.163, G3 = 3.0±0, *p*<0.05).

**Table 2:** Score evaluation of postero-lateral fusion via radiographs (n = 10 per each group).

	X-ray (Number)		
score	G1	G2	G3
0	2	0	0
1	8	0	0
2	0	6	0
3	0	4	10
Mean±SD:	0.8±0.133	2.4±0.163	3.0±0

There were significant differences among three groups (G2 *vs* G1,*p*<0.05;G3 *vs* G1, *p*<0.05; G3 *vs* G2, *p*<0.05). It indicated the collagen scaffolds had a positive effect on spine fusion and CBD-BMP-2 accelerated the fusion.

**Figure 5:**
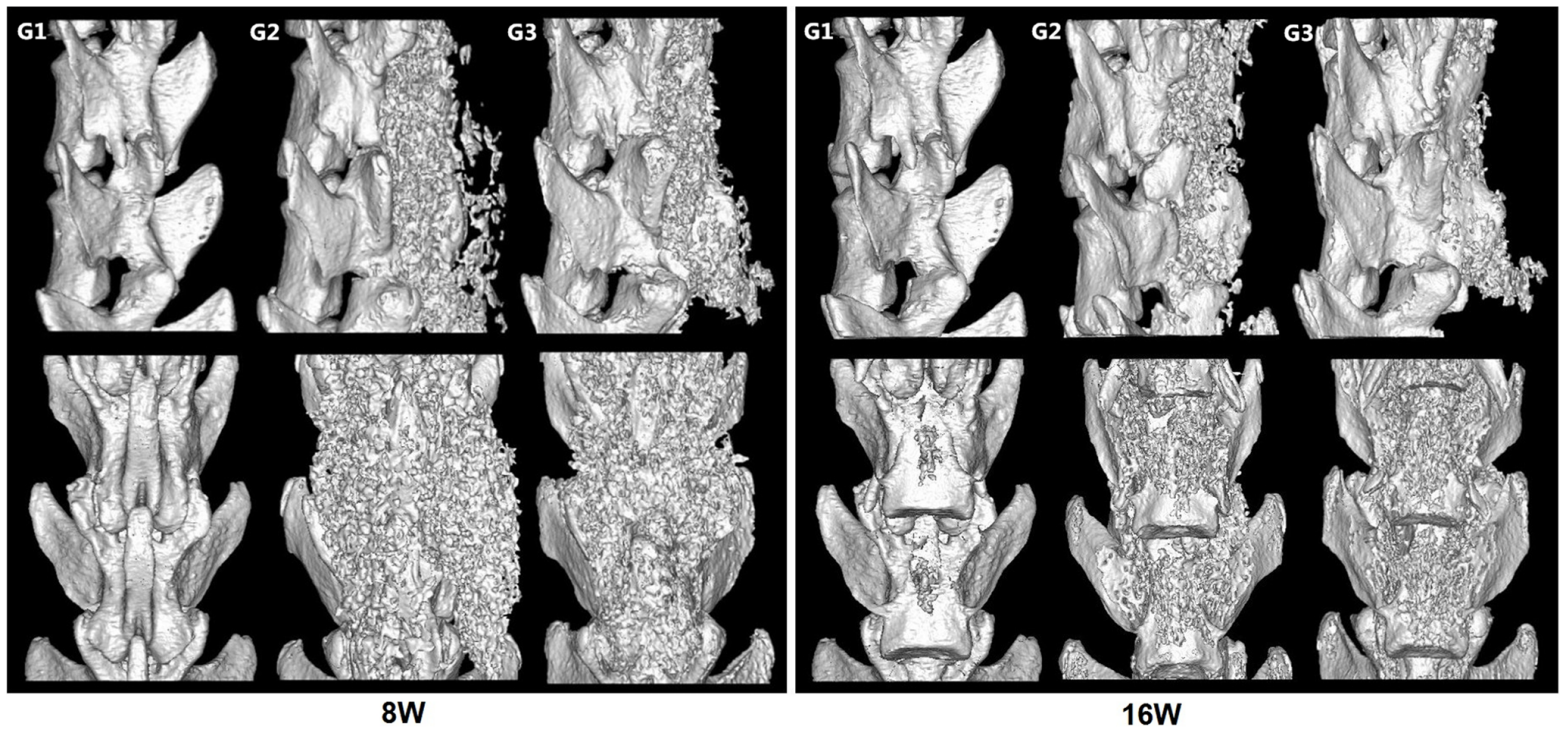
Representative 3D reconstruction images of Micro-CT images. 8W: The images of 8 weeks after the implantation showed that obvious new bone formed in G2 and G3 while a similar phenomenon did not appear in G1. 16W: The images of 16weeks after the implantation showed that the spines of all the rats exhibited considerable new bone formation in G3 and fusion masses were significantly larger and denser than G2. There was still no new bone found in G1.

**Figure 6:**
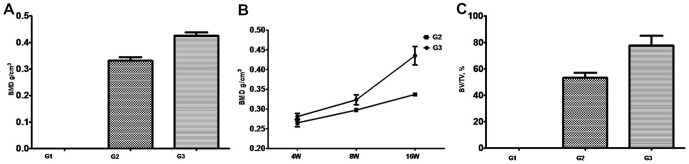
Bone mineral density (BMD) analysis by Micro-CT. A: BMD analysis at 16 weeks after surgery. B: The BMD at different time points. C: The ratio of new bone volume (BV) to tissue volume (TV) at 16weeks. Data are expressed as the mean±SD (n = 10 per each group).

Micro-CT was also used for bone formation assessment. BMD in Fig.5 showed that the gaps between the L4 and L5 transverse processes were filled with new bone tissues in G2 and G3 groups, but the similar phenomenon did not appear in G1 group. The volume of new bone formation was much larger in G3 compared to G2. Moreover, the new bone formed in the area where the scaffolds were implanted, but did not migrated or had ectopic osteogenesis, suggesting that collagen scaffolds retained the migration of CBD-BMP-2. The BMD analysis of the spines demonstrated substantial bones emerged between the transverse processes in G3 group. A statistical analysis of BMD showd a significant difference among three groups (Fig. 6A, G1: 0g/cm^3^, G2: 0.3337±0.0025g/cm^3^, G3: 0.4353±0.0234g/cm^3^. *p*<0.05). Furthermore, we found that the increased BMD between 4 and 8 weeks was less than that between 8 and 16 weeks (Fig. 6B), which may be a result of collagen degradation. We randomly selected a cylindrical area of 1mm in diameter and 100 layers in thickness from the scanograms of Micro-CT to quantify the ratio of bone volume to tissue volume, representing the quantity of new bone formation. The results showed that the collagen/CBD-BMP-2 composites led to much more new bone formation than collagen alone (Fig. 6C, G3: 77.68±14.76%, G2: 53.28±6.499%, *p*<0.05). Meanwhile, the degradation of collagen scaffolds was clearly observed at 16 weeks after the surgery compared to the one at 8 weeks.

### New bone formation was assessed by histologic analysis

**Figure 7:**
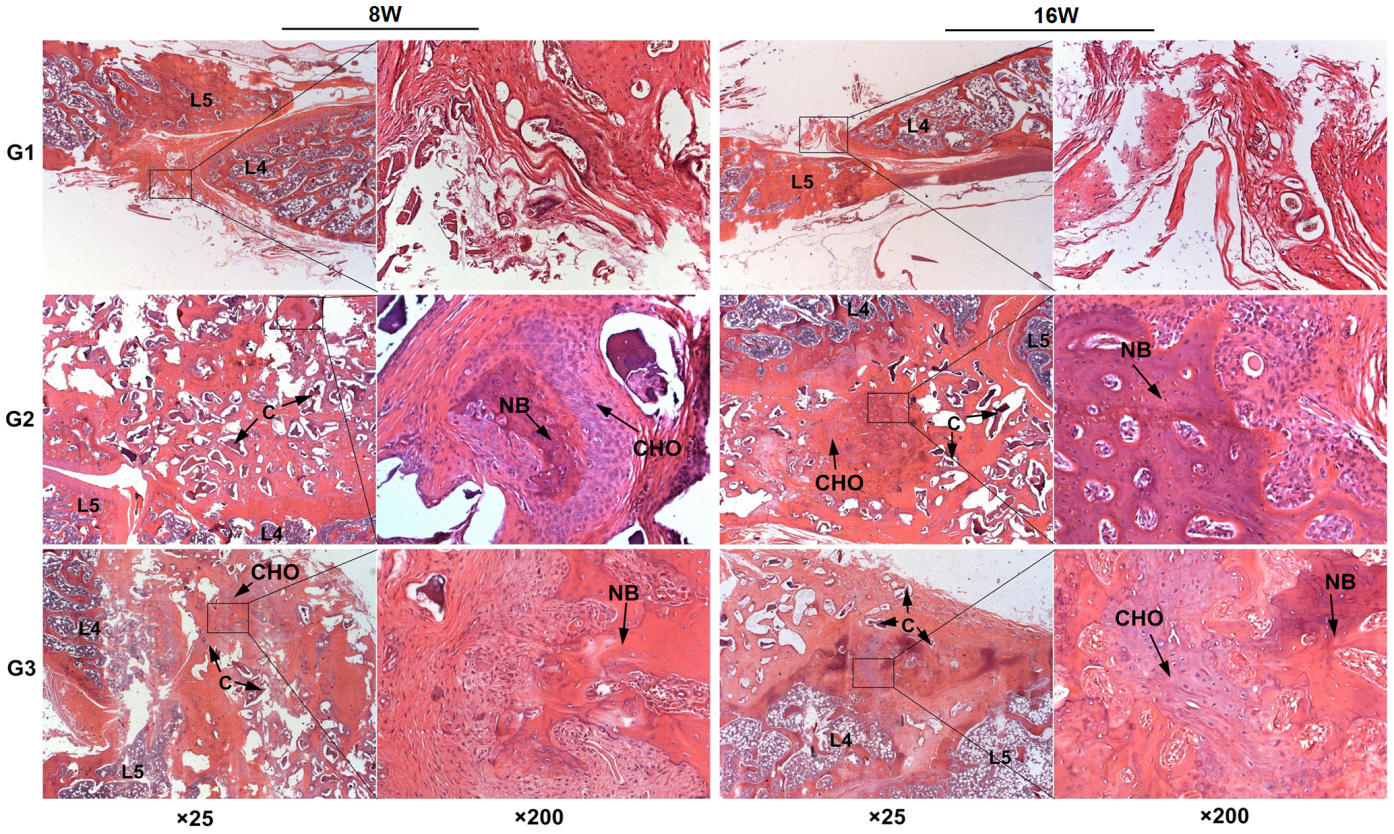
The H&E staining for L4–L5 posterolateral space in rats. There were plenty of trabeculae between spongy bone which mean a fusion in G2 and G3 and no new bone were found in G1 (C: incompletely degraded collagen scaffolds, CHO: chondrocyte, NB: new bone).

H&E staining provided an objective assessment on collagen degradation and bone formation. As shown in Fig.7, trabeculae were barely observed in G1 group, while a great quantity of trabeculae were found in G2 and G3 groups. Chondrocytes and osteoblasts around the new bone area were also detected, indicating an active process of new bone formation. The amount of trabeculae and osteoblasts in G3 group was much greater compared to G2 group. Same as previous study [12], [13], the partially degraded collagen scaffolds were also observed (Fig. 7).

## Discussion

In recent years, an increasing number of patients have been receiving spine fusion operations to prevent further instability and insult to the cord. Autogenous iliac crest was used as a substitute to provide the osteoinductive and osteoconductive effect for the new bone formation in the traditional spinal fusion procedures. But the volume of bone harvested from the donor site is limited and the harvest of autograft may be associated with various complications [26], [27]. Allografting does not have these complications. However, it increases the risk of disease transfer. An attractive strategy is to combine osteoinductive cytokine with osteoconductive vehicle as an alternative to stimulate new bone formation [9].

BMP-2 has been shown to be successful in achieving spinal fusion in animals and humans. Subsequent pre-clinical studies have shown that the interbody spinal fusion rate in BMP-2 group was almost 100% while only 30%–50% fusion rates in the autograft controls and a similar outcome were obtained in clinical studies [28], [29]. Favorable results of BMP-2 in improving the rates of interbody spinal fusion stimulated the increased use of BMP-2 in analogous spine procedures. According to a report, by 2007, more than 50% of primary anterior interbody lumbar fusion (ALIF), 43% of posterior lumbar interbody fusion (PLIF)/transforaminal lumbar interbody fusion (TLIF), and 30% of posterolateral lumbar fusion (PLF) were reported to use BMP-2 [29]. These reports suggest that BMP-2 has been accepted as an effective inducing factor of osteogenesis.

However, controversies are still associated with the clinical applications of BMP-2. A number of reported complications include osteolysis, seroma with radiculopathy, soft-tissue swelling, increased infections, cage subsidence or migration and so on [30]. Bae HW et al. provided strong support that excessive BMP-2 could cause trachea edema, and even death. Some of patient developed retroperitoneal hematoma (7.2%) [31]. Devine JG et al. reviewed the cancer risk of rhBMP-2 used in spine fusion as published and came to a conclusion that cancer risk with BMP-2 may be dose dependent and a higher dose cause a higher risk of cancer, even when collage scaffold are used to slow the release of BMP-2, while the risk of cancer reduced to the level of control groups in a dose of BMP-2 (4.2–4.8mg) [32]. Furthermore, the excessive dosage of BMP-2 could create other potential risks, such as excessive bone formation and adverse immune responses [33]. Thus, it is critical to maintain the therapeutic function with the dosage of BMP-2 as low as possible in clinical application.

It has been well established that BMP-2 requires a suitable delivery vehicle to be effective. In 2002, the Federal Drug Administration (FDA) approved a device called INFUSE, which contained a porous resorbable collagen sponge to retain the BMP-2 [34], [35]. However, the open porous structure of the collagen carrier failed to reduce the dosage and some side effects of BMP-2. Spilled cytokines cause not only significant waste but also many kinds of sequelae [32]. Further investigations focus on improving the BMP-2 efficacy in INFUSE. A great number of new biomaterials have emerged in recent years such as bacterial cellulose, PEGylated fibrinogen, hydroxyapatite (HA), supramolecular nanofibers [6], [8], [10]. In addition to the ability of sustaining release of the BMP-2 for sufficient time, these biomaterials also can partially avoid the complications caused by BMP-2. Thus, to reduce the negative effect, we introduced collagen scaffolds incorporated with CBD-BMP-2 to enhance posterolateral intertransverse process fusion in rats.

In this study, we prepared the collagen scaffolds from bovine aponeurosis. As previously described, the processed material showed good cell compatibility and low immunogenisity[14]. Meanwhile, the collagen scaffolds were characterized by a proper porosity (65%) and micropores size (100–300 µm), which showed excellent osteoconduction [31]. The data of histology and imageology showed that new bone formation was appeared in collagen-treated group, indicating that collagen scaffolds has clear osteoconduction and play a positive role in spine fusion.

Although the collagen scaffolds can retain the BMP-2, partially avoiding problems of the complications caused by the high focal concentration of BMP-2, some studies have reported the phenomena of burst release upon initial implantation. Here we modified the BMP-2 by adding a collagen binding domain (CBD). By the site-specific combination, the modified BMP-2 could be incorporated to the collagen scaffolds and achieve a sustained release. In our previous and present study, the CBD-BMP-2 showed a higher affinity to collagen scaffolds than that of the commercial BMP-2. Without burst release and quick degradation, it suggested that engineered CBD-BMP-2 be administrated at a lower dose compared to traditional BMP-2. The INFUSE contains 1.5 mg BMP-2 per milliliter, which exceed the physiological levels of BMP-2 (1 µg/kg). In clinic, it is also generally recognized that the average BMP-2 dosage per implant site was 12–40 mg, and the BMP-2 concentration showed a gradual decrease over time [24], [36], [37]. To enhance posterolateral intertransverse process fusion in rats, we administrated CBD-BMP-2 at a dose of 20 µg per milliliter collagen scaffold, which was much less than the commercial INFUSE. As we respected, the group with collagen scaffolds incorporated CBD-BMP-2 had better results than with collagen scaffolds alone, including higher fusion efficiency, higher bone mineral density (BMD) and more bone trabecular formation. We speculated that the sustained-release system developed here reduced the BMP-2 burst release because of site-specific collagen binding domain. It was benefited to avoid of heterotopic bone formation in the clinical application. Meanwhile, CBD-BMP-2 was released into surrounding environment during the collagen scaffold degraded. It was helpful for BMP-2 being an osteoinduction factor as long as possible. However, further investigations on controlling the BMP2 release via the collagen scaffolds are necessary.

We did not observe overgrowth and heterotopic bone formation in the experiments. All rats survived without infection and did not suffer inflammation at the site of implantation. Our results indicated that CBD-BMP-2 can stimulate the new bone formation and promote spine fusion without any detectable side effects of BMP-2. It suggested that combination delivery could be an alternative candidate in spine fusion with decreased dramatically side effects caused by high dose of BMP-2. This advantage needs investigation and verification in future application.

However, there still have many variables which were not taken into consideration. The fusions in SD rats have been limited to posterolateral fusion and can not provide enough iliac crest bone for posterolateral fusion as controls. Larger animals can offer advantages for experiments that study internal fixation devices and biomechanics[38]. So we need larger animal model to further confirm our results. Meanwhile tetrapods like rats can not reflect the condition of the spine in bipedality because lumbars are not the highest load bearing region[39]. Methods used in our experiments need to be further improved, such as the BMD date should be optimized to reflect the bone density. In addition, we need further to compare the effects of different dose of CBD-BMP-2 on spine fusion in vivo. All the problems would be solved in future.

## Conclusion

Collagen scaffolds loaded with CBD-BMP-2 enhanced a posterolateral intertransverse process fusion. With site-specific collagen binding domain, BMP-2 could be adhensive to carries for a longer time than commercial BMP-2, which suggested a potential delivery system to promote bone regeneration and to increase the probability of spine fusion.
